# Synthesis, Cytotoxic and Genotoxic Evaluation of Drug-Loaded Silver Nanoparticles with Mebeverine and Its Analog

**DOI:** 10.3390/ph18030397

**Published:** 2025-03-12

**Authors:** Mihaela Stoyanova, Miglena Milusheva, Milena Georgieva, Penyo Ivanov, George Miloshev, Natalia Krasteva, Kamelia Hristova-Panusheva, Mehran Feizi-Dehnayebi, Ghodsi Mohammadi Ziarani, Kirila Stojnova, Slava Tsoneva, Mina Todorova, Stoyanka Nikolova

**Affiliations:** 1 Department of Organic Chemistry, Faculty of Chemistry, University of Plovdiv, 4000 Plovdiv, Bulgaria; stoyanova@uni-plovdiv.bg (M.S.); or miglena.milusheva@mu-plovdiv.bg (M.M.); 2 Department of Bioorganic Chemistry, Faculty of Pharmacy, Medical University of Plovdiv, 4002 Plovdiv, Bulgaria; 3 Laboratory of Molecular Genetics, Epigenetics and Longevity, Institute of Molecular Biology “R. Tsanev”, Bulgarian Academy of Sciences, 1113 Sofia, Bulgaria; milenageorgy@gmail.com (M.G.); p.ves.iv@gmail.com (P.I.); karamolbiol@gmail.com (G.M.); 4 Institute of Biophysics and Biomedical Engineering, Bulgarian Academy of Sciences, 1113 Sofia, Bulgaria; natalia.krasteva@yahoo.com (N.K.); kameliahristova@abv.bg (K.H.-P.); 5 Department of Organic Chemistry, Faculty of Chemistry, Alzahra University, Tehran P.O. Box 19938-93973, Iran; m.feizi@alzahra.ac.ir (M.F.-D.); gmohammadi@alzahra.ac.ir (G.M.Z.); 6 Department of General and Inorganic Chemistry with Methodology of Chemistry Education, Faculty of Chemistry, University of Plovdiv, 4000 Plovdiv, Bulgaria; stoynova@uni-plovdiv.bg; 7 Department of Analytical Chemistry and Computer Chemistry, University of Plovdiv, 4000 Plovdiv, Bulgaria; slava.tsoneva@uni-plovdiv.bg

**Keywords:** silver nanoparticles, cytotoxicity, genotoxicity, in vitro, in silico, molecular docking, irritable bowel syndrome, mebeverine

## Abstract

**Background:** Irritable bowel syndrome (IBS) is a prevalent gastrointestinal disorder with a complex pathogenesis that necessitates innovative therapeutic approaches for effective management. Among the commonly used treatments, mebeverine (MBH), an antispasmodic, is widely prescribed to alleviate IBS symptoms. However, challenges in delivering the drug precisely to the colonic region often hinder its therapeutic effectiveness. To address this limitation, silver nanoparticles (AgNPs) have emerged as promising drug delivery systems, offering unique physicochemical properties that can enhance the precision and efficacy of IBS treatments. **Objectives:** This study aimed to synthesize AgNPs as drug delivery vehicles for MBH and a previously reported analog. The research focused on evaluating the cytotoxic and genotoxic effects of the AgNPs and forecasting their possibly harmful effects on future sustainable development. **Methods:** AgNPs were synthesized using a rapid method and functionalized with MBH and its analog. The nanoparticles were characterized using different techniques. Cytotoxicity and genotoxicity were evaluated in vitro. Additionally, in silico docking analyses were performed to explore their safety profile further. **Results:** In vitro assays revealed concentration-dependent cytotoxic effects and a lack of genotoxic effects with MBH-loaded AgNPs. A molecular docking simulation was performed to confirm this effect. **Conclusions:** The study underscores the potential of AgNPs as advanced drug delivery systems for safe and significant therapeutic implications for IBS. Future in vivo and preclinical investigations are essential to validate the safe range of exposure doses and evaluation standards for assessing AgNPs’ safety in targeted and personalized medicine.

## 1. Introduction

Irritable bowel syndrome (IBS) is a prevalent gastrointestinal disorder characterized by symptoms such as abdominal pain, bloating, and altered bowel habits, including diarrhea and constipation. The exact cause of IBS remains unclear, but it is believed to involve a combination of factors, including gut–brain interactions, motility disturbances, and visceral hypersensitivity [1,2]. This illness affects 5% and 10% of the general population [3,4]. Management strategies typically focus on alleviating symptoms and improving patients’ quality of life. Although the pathogenesis of irritable bowel syndrome remains unclear, it is known that abnormal gut–brain connection results in visceral hypersensitivity, abnormal CNS processing, and motility issues [5,6].

**Figure 1:**
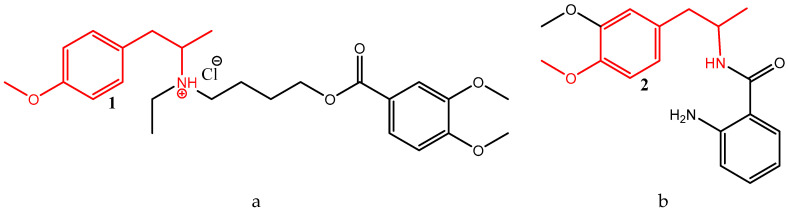
Structure of (**a**) MBH hydrochloride (**1**); (**b**) MBH analog (MA, **2**). Common structure in red.

Mebeverine (MBH (**1**), Figure 1) is an antispasmodic commonly prescribed to manage IBS symptoms. It functions by directly relaxing the smooth muscles of the gastrointestinal tract, thereby reducing spasms and associated discomfort [7]. Unlike other antispasmodics, MBH does not exhibit typical anticholinergic side effects, such as dry mouth or blurred vision, making it a favorable option for many patients [8]. Consequently, MBH is widely used as a relaxant agent for treating gastrointestinal spasmodic disorders such as abdominal pain, intestinal disturbances, and bowel discomfort [9]. The main problem facing researchers in IBS treatment is targeting the medications directly to the colonic region [10]. Nanoparticles (NPs), on the other hand, have become ground-breaking instruments in various medical applications due to their unique qualities, such as increased surface reactivity, size control, and the capacity to be functionalized with therapeutic agents. Remarkably, they are valuable in drug delivery, diagnostics, cancer therapy, and gene therapy. To maximize safety and efficacy, NPs may enhance the stability and solubility of encapsulated cargos, facilitate transport across membranes, and extend circulation periods [11,12]. These factors have led to a large amount of NP research, which has produced encouraging outcomes in small animal models and in vitro [13].

Silver and gold nanoparticles (NPs) have garnered significant interest as drug delivery systems due to their unique physicochemical properties, such as high surface area-to-volume ratio, ease of functionalization, and excellent stability. In particular, silver nanoparticles (AgNPs) have demonstrated versatility in delivering a wide range of therapeutic agents. AgNPs enhance drug solubility, protect sensitive compounds from degradation, and provide controlled and sustained release at targeted sites. They have further been explored for the delivery of antibiotics, where they improve antimicrobial efficacy and help overcome resistance by targeting bacterial biofilms and disrupting cell membranes [14]. Additionally, AgNPs are used to deliver anti-inflammatory drugs, which enhance drug stability and provide targeted action at inflamed tissues, minimizing systemic side effects [15,16]. AgNPs have been incorporated into drug delivery systems in wound healing to promote tissue regeneration while simultaneously preventing infections, thanks to their inherent antimicrobial properties [17,18]. They are also employed for the topical delivery of corticosteroids and growth factors, improving the therapeutic outcomes for skin disorders and chronic wounds [19].

AgNPs have been utilized in gene therapy as carriers for nucleic acids, such as siRNA and plasmid DNA. By protecting these biomolecules from enzymatic degradation and facilitating their cellular uptake, AgNPs enable efficient gene silencing or expression in target cells [20]. They have additionally shown promise in the delivery of anti-diabetic drugs, where their functionalization with specific ligands enhances the targeting of pancreatic β-cells or improves glucose regulation [21]. Moreover, their role in delivering antiviral agents has been investigated, particularly for enhancing the stability and bioavailability of drugs targeting viral replication processes [22]. The ability to cross biological barriers, including the blood–brain barrier, makes this particular type of nanoparticle an attractive option for central nervous system (CNS) drug delivery, where it can enhance the bioavailability of therapeutic agents for treating neurological disorders [23]. These multifunctional capabilities of silver nanoparticles extend beyond their intrinsic biological activities, making them valuable tools for the precise and effective delivery of a diverse array of therapeutic agents across different medical applications.

On the other hand, size, surface chemistry, exposure techniques, and exposure duration are important factors that affect AgNP tissue distribution paths [24]. Individuals may be exposed to AgNPs by a variety of routes, such as blood circulation (intravenous injection), oral administration (gastrointestinal tract), inhalation (respiratory tract), and skin contact [25]. In vivo, injected AgNPs have a broad tissue distribution and a brief circulation duration through tail veins [16]. Although larger AgNPs may concentrate more in the spleen than in other organs, the liver is the primary target organ, followed by the spleen, lungs, and kidneys. It is interesting to note that blood levels of Ag stay elevated for up to six days following injection, after which they begin to decline. This shows that AgNPs may undergo time-dependent degradation and removal [26].

Additionally, a study on the toxicity of inhaled AgNPs found that their distribution to the liver and lungs causes inflammatory reactions like chronic alveolar inflammation and inflammatory cell infiltration [27]. Furthermore, migration to the gastrointestinal tract’s acidic environment aids in dissolving AgNPs into silver ions when exposed orally. AgNPs also impact inflammation, apoptosis, and the expression of biochemical indicators of hepatotoxicity, including serum cholesterol and alkaline phosphatase [28,29,30]. Additionally, there were no discernible changes in the health condition of workers exposed to low levels of silver dust or soluble silver at threshold limit values [31]. A safe range of exposure levels and evaluation criteria for determining AgNP safety must be established. Assessing the biological impact is the primary goal of nanotoxicity, which is crucial for understanding mechanisms and predicting the potentially detrimental effects of AgNPs for their future development.

Here, we aim to address the challenges of targeted drug delivery for IBS by synthesizing AgNPs functionalized with MBH and MA. We evaluate these drug-loaded nanoparticles’ cytotoxic and genotoxic profiles through in vitro assays and explore their molecular interactions using in silico docking analyses. This approach paves the way for sustainable and personalized medicine applications, offering innovative strategies to enhance therapeutic efficacy for IBS.

## 2. Results and Discussion

To explore the potential biomedical applications of silver nanoparticles (AgNPs), it is essential to develop synthesis and functionalization strategies that ensure efficacy, safety, and biocompatibility. Chemical reduction is a traditional procedure that entails dissolving a silver salt in a liquid phase to react with a reducing agent. To enhance biological activity while minimizing toxicity, we employed a chemical reduction as a one-pot synthesis method that leverages fructose’s reducing and capping properties [16,32]. This approach facilitated the efficient formation of AgNPs, which were subsequently functionalized with MBH (**1**) and its analog MA (**2**) (Figure 1). AgNPs were selected due to their diverse biological activities, including antimicrobial, anti-inflammatory, and antioxidative effects [33,34,35,36,37,38,39,40]. Beyond these properties, their protective effect on the gastrointestinal tract is particularly significant [41,42]. Recent research highlights their potential to shield the gut lining by reducing oxidative stress, alleviating inflammation, and restoring gut microbiota balance [43]. This protective action extends to conditions such as inflammatory bowel disease (IBD) and gastric ulcers, where AgNPs may play a supportive therapeutic role. Additionally, AgNPs have been shown to mitigate damage caused by pathogens and chemical irritants, thus contributing to maintaining gastrointestinal health [15].

Using fructose as a reducing and capping agent was critical for achieving the desired properties of the synthesized AgNPs. At the boiling temperature of water (100 °C), Ag+ ions were rapidly reduced to their ground state, resulting in the formation of fructose-capped AgNPs (**3**) [44]. These nanoparticles were explicitly chosen for their lower toxicity than other capping agents [45,46], making them more biocompatible and suitable for medical research and practice applications.

### 2.1. Particle Size and Characteristics of AgNPs with MBH (***4***) Compared to AgNPs with MA (***5***)

**Figure 2:**
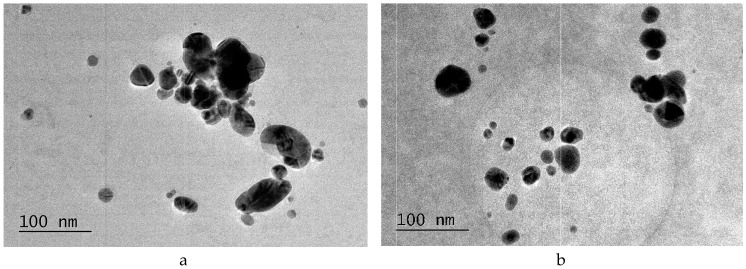
TEM images of (**a**) AgNPs with MBH (**4**) and (**b**) AgNPs with MA (**5**).

To establish the size and shape of the AgNPs, TEM, DLS, and zeta potential were used. TEM images also indicate the shape and size of the nanoparticles and illustrate the individual nanoparticles. The TEM images confirmed the synthesis of smaller spherical particles with different sizes in the range of 3 to 10 and 90 to 95 nm for (**4**) (Figure 2a) and between 84 and 95 nm size for (**5**) (Figure 2b).

AgNPs were obtained with controllable size due to carbohydrate assistance in the presence of [Ag(NH_3_)_2_]^+^ [47]. It is well known that irrespective of the approach, biomolecules are involved in the reduction of silver nanoparticles and in an interaction with the upper face of silver, which is their initial connection of originating particles [48,49]. The carbohydrate coating of AgNPs decreases the agglomeration rate and size. Many groups are likely absorbed on the surface of silver due to covalent bonding to oxygen and nitrogen for the complex formation of silver [50]. The dynamic surface site of the AgNPs is determined based on the particle size, shape, and accumulation rate. The reaction conditions such as temperature, pH, extract volume, reactant concentration, and time describe the growing particles’ size and gradation and influence the silver accumulates’ shape and size [51].

**Figure 3:**
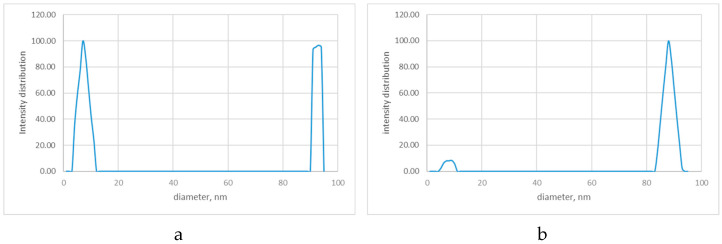
Dynamic light scattering histograms of (**a**) AgNPs with MBH (**4**) and (**b**) AgNPs with MA (**5**).

The median average size of the obtained particles was confirmed by DLS (Figure 3). The histograms of both samples (**4**) and (**5**) displayed a bimodal particle size distribution, suggesting the presence of aggregates.

The zeta potential of (**4**) was −19.19 mV, and that of (**5**) was −13.64 mV. Both samples’ negative charge of the zeta potential refers to carboxylic groups of gluconic acid obtained when Ag^+^ is reduced to Ag°. Carboxylic acids, obtained in the oxidation of sugars, provide a negative surface charge density to counteract the van der Waals forces responsible for particle coalescence. Self-assembled carboxylic acids ensure dense coating on the metal surfaces and stabilize them [52].

### 2.2. UV-Vis and FTIR Spectra of AgNPs with MBH (***4***) and AgNPs with MA (***5***)

**Figure 4:**
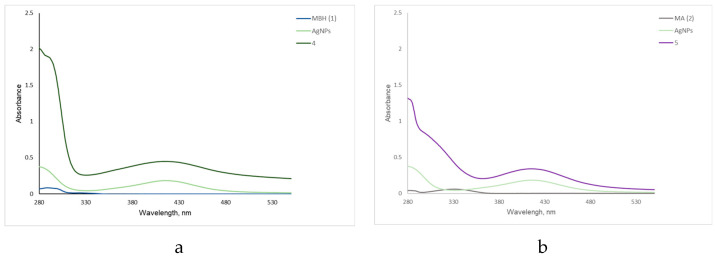
Absorption spectra of (**4**) and (**5**): (**a**) MBH (**1**)—blue; AgNPs (**3**)—light green; AgNPs with MBH (**4**)—dark green; (**b**) MA (**2**)—grey; AgNPs (**3**)—light green; AgNPs with MA (**5**)—purple.

The synthesis of the Ag NPs in an aqueous solution was monitored by recording the absorption spectra in the wavelength range of 190–600 nm (Figure 4). UV/Vis absorption spectroscopy is a crucial tool for investigating AgNPs in suspension. Each type of AgNP exhibits intriguing optical properties associated with surface plasmon resonance [53]. The time-dependent UV/Vis absorption spectra of AgNPs with MBH **4** (Figure 4a) and AgNPs with MA **5** (Figure 4b) are presented. Figure 4 shows that MBH (a) and MA (b) have absorption maxima at 229 nm and 225 nm, respectively, and fructose has an absorption peak at 189 nm. The indicator for the presence of AgNPs is the SPR characteristic band at 415 nm. The position of this band between 350 and 420 nm indicates the spherical shape of the particles [54].

The SPR peak is broadening in both spectra (Figure 4a,b). The peak of (**4**) is symmetrical, which indicates the low degree of aggregation of the NPs [16,55]. A slight asymmetry is observed in the SPR peak of (5). The reasons for the asymmetry are different; it may be due to aggregation [56] or the existence of tautomeric forms [32]. The most likely explanation is the potential for intramolecular hydrogen bonding between the primary and secondary amino group in (**2**), which would result in forming a six-membered ring. We eliminate the potential for aggregation since the carbohydrate utilized to make the nanoparticles also helps stabilize them [57].

The deposition of (**1**) and (**2**) on AgNPs’ surface, forming (**4**) and (**5**), has a negligible effect on the position of the SPR peak—422 nm (**4**) and 420 nm (**5**)—suggesting no significant alteration in their optical properties.

**Figure 5:**
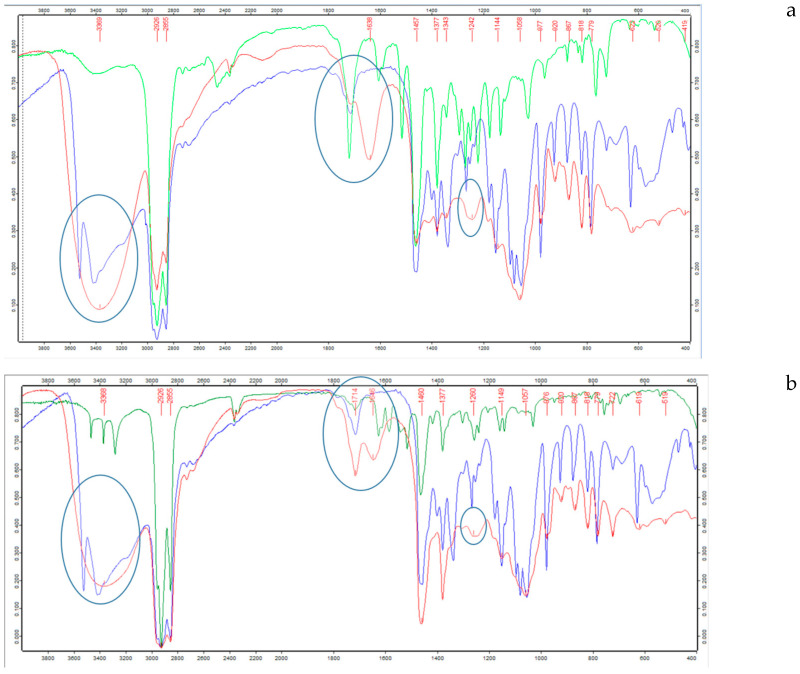
FTIR spectra of (**4**) and (**5**): (**a**) fructose—blue; AgNPs with MBH (**4**)—red; MBH (**1**)—green; (**b**) fructose—blue; AgNPs with MA (**5**)—red; MA (**2**)—green.

The majority of the distinctive bands of fructose are presented in the FTIR spectra of AgNPs with MBH (**4**) and AgNPs with MA (**5**) (Figure 5), along with a few extra and modified bands. In the FTIR spectrum of fructose (blue), the broad peak with a maximum at approximately 3417 cm^−1^ and its corresponding sharp band at 3524 cm^−1^ represent the stretching vibrations of the hydroxyl groups (-O-H), which are H-bonded and free hydrogen bonds, respectively. Additionally, the O-H stretching vibration mode linked to intramolecular and intermolecular hydrogen bonding was identified as the strong, broadband source at 3369–3368 cm^−1^ in (**4**) and (**5**), respectively.

The 1058/1057 cm^−1^ peak can be assigned to C-O vibrations. The significant peak at 1713–1714 cm^−1^ is due to the C=O stretching of the -COOH group of the fructose unit. Compared to the FTIR spectrum of fructose, the corresponding peaks in the spectrum of (**4**) and (**5**) exhibit visible changes for the peaks of the COO– (around 1638 cm^−1^) and O-H groups (around 3369 cm^−1^). These can be considered an indication for the synthesis and stabilization of the AgNPs due to the responsible carboxyl and hydroxyl groups [32,44,58]. The weak broadband at 2104 cm^−1^ in both the (**4**) and (**5**) spectrum can be assigned to –CO-Ag linkages [59].

### 2.3. Effect of (***1***) to (***5***) on HepG2 Cell Morphology

The rapidly expanding application of nanoparticles in daily life raises concerns regarding their safety. AgNP risk assessments are intended to investigate biological consequences, potential causes, and practical strategies to reduce adverse effects. Less than 10 nm of AgNPs may cause mutagenicity and is linked to genotoxicity due to oxidative stress-induced damage to DNA and chromosomes [60,61]. Elemental mapping of individual cells and transmission electron microscope pictures demonstrated that AgNPs can translocate to the nucleus and harm chromosomes and DNA [62,63,64]. Therefore, our primary goal was to assess the cytotoxic and genotoxic effects of synthesized AgNPs.

**Figure 6:**
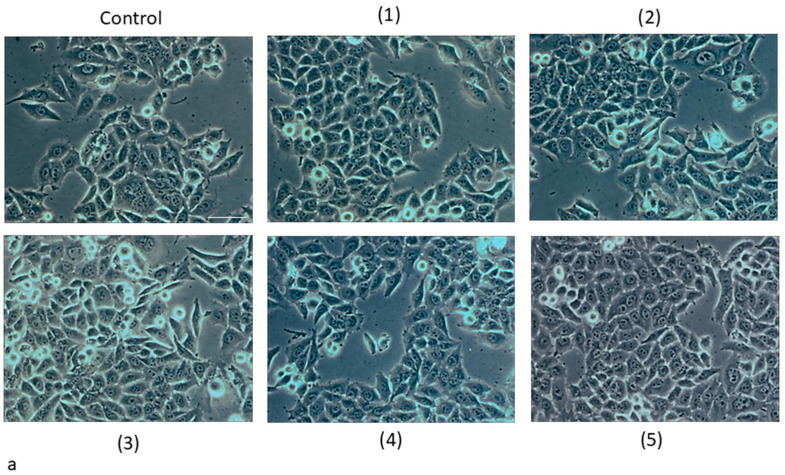

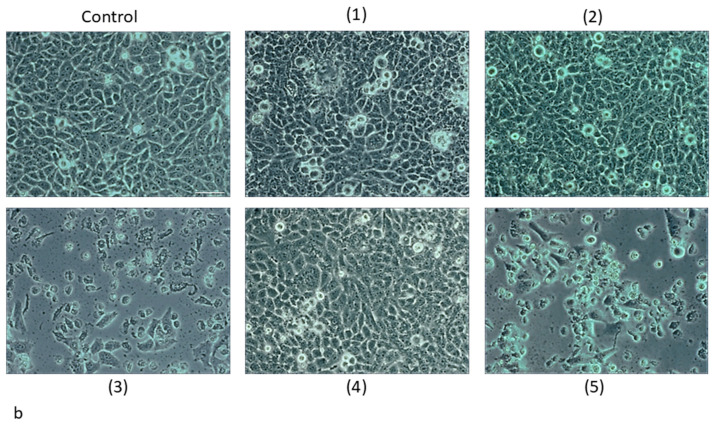
Phase-contrast micrographs of HepG2 cells treated for 24 h (**a**) and 72 h (**b**) with substances (**1**) to (**5**). Morphological changes in the cells were monitored to evaluate potential cytotoxic effects and alterations in cell structure. Non-treated HepG2 cells were used as the control group to establish baseline morphology and ensure the reliability of the observations. Images were captured at a magnification of 20×, with a scale bar of 50 µm for reference.

A sign of NPs’ cytotoxicity is the alteration in normal cell morphology following treatment with NPs. Therefore, in this study, we assessed HepG2 cell morphology using phase-contrast microscopy at 24 h and 72 h after treatment with the tested substances. HepG2 cells were chosen for this investigation because they are widely recognized as excellent models for testing bioactivity. Their well-characterized metabolic and physiological properties closely mimic those of human hepatocytes, making them invaluable for studying the bioeffects and cytotoxicity of various substances [65]. At 24 h, only cells treated with substance (**3**) exhibited slight morphological changes, characterized by a more elongated cell shape. However, a more pronounced alteration in morphology was observed at 72 h. HepG2 cells treated with substances (**3**) and (**5**) displayed distinct apoptotic features. Compared to the untreated control cells, those treated with (**3**) and (**5**) exhibited irregular and fragmented morphology, with most cells detaching and dying. In contrast, untreated HepG2 cells maintained a healthy, polygonal shape with more regular dimensions and formed tightly packed monolayers (Figure 6).

### 2.4. Effect of (***1***) to (***5***) on HepG2 Cell Viability

**Figure 7:**
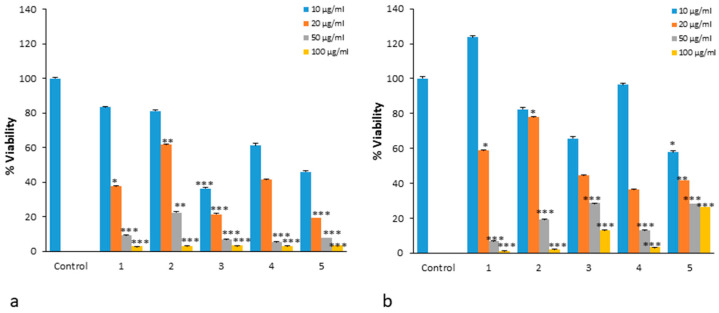
Analysis of cell viability using the WST-1 assay following treatment with substances (**1**) to (**5**): (**a**) at 24 h and (**b**) at 72 h. Non-treated HepG2 cells served as the control group for baseline comparison. Data are presented as mean ± SEM, with n = 3 independent experiments. Statistical significance is denoted by asterisks, with * *p* < 0.05, ** *p* < 0.01, and *** *p* < 0.001 indicating significant differences between the treated and control samples. These results provide insight into the cytotoxic effects of the tested substances over time.

To further evaluate the cytotoxicity of the tested substances, HepG2 cells were treated with increasing concentrations (ranging from 10 μg/mL to 100 μg/mL) for 24 and 72 h, and the cell viability was assessed using the WST-1 assay. The results demonstrated a concentration-dependent decrease in cell viability at both time points, as depicted in Figure 7 and summarized in Table 1. At 24 h, substance (**3**) (AgNPs) exhibited the most significant cytotoxicity, reducing cell viability to 36.9% at the lowest concentration (10 μg/mL). This was followed by substance (**5**) (AgNPs with MA), which reduced viability by 57.7%, and substance (**4**) (AgNPs with MBH), with a reduction of 38.81%. Substance (**2**) (the analog) and substance (**1**) (MBH) showed the least cytotoxic effects, with reductions of 18.83% and 16.46%, respectively. All substances exhibited significant cytotoxicity at the highest concentration (100 μg/mL), reducing cell viability to 2.89–3.48%. The IC_50_ values calculated from these data confirmed the cytotoxicity order at 24 h as (**3**) > (**5**) > (**4**) > (**2**) > (**1**).

Interestingly, at 72 h, a shift in the cytotoxicity profiles was observed. Substance (**1**) displayed a stimulatory effect on cell viability at the lowest concentration (10 μg/mL), indicating a potential adaptive cellular response. However, at higher concentrations (50 and 100 μg/mL), it became the most cytotoxic, showing a marked decrease in cell viability. Substance (**2**) alone continued to exhibit relatively low cytotoxicity, but when loaded onto AgNPs as substance (**5**), it became significantly more toxic, surpassing the cytotoxicity of (**3**) as plain AgNPs. At 72 h, the order of toxicity based on IC_50_ values shifted to (**5**) > (**3**) > (**2**) > (**4**) > (**1**).

These results seem to be supported by other authors who found that a significant reduction in cell survival was detected after 48 h of incubation following AgNP treatment [66].

These findings align with our hypothesis that AgNPs could be a viable delivery vehicle for MBH and MA, as they enhance the therapeutic agents’ bioavailability while maintaining manageable cytotoxicity levels. The data also highlight the mitigated cytotoxicity of nanoparticle formulations compared to plain AgNPs, reinforcing their potential as effective and safe drug delivery systems. Notably, while AgNPs alone exhibited pronounced cytotoxic effects, their combination with MBH (**4**) or MA (**5**) effectively reduced the inherent toxicity of the nanoparticles, supporting their use in therapeutic applications.

The higher cytotoxicity observed in HepG2 cells treated with AgNPs (**3**) and AgNPs with MA (**5**) can be attributed to multiple factors. The small size and high surface area of AgNPs enhance cellular uptake, increasing the active agents’ intracellular concentrations and amplifying their cytotoxic effects [67]. Additionally, combining AgNPs with MA in substance (**5**) likely results in a synergistic interaction, further potentiating its cytotoxicity. AgNPs also activate cellular stress pathways, including DNA damage and mitochondrial dysfunction, contributing to enhanced cell death. Moreover, the sustained release of MA from the nanoparticle matrix prolongs its bioactivity, maintaining a continuous cytotoxic effect [68,69,70]. These mechanisms collectively explain the pronounced cytotoxicity observed with substances (**3**) and (**5**), particularly at higher concentrations and extended exposure times. Despite these findings, the formulations demonstrate a promising balance of efficacy and safety, supporting their potential use as targeted therapeutic delivery systems.

### 2.5. Reactive Oxygen Species (ROS) Production

**Figure 8:**
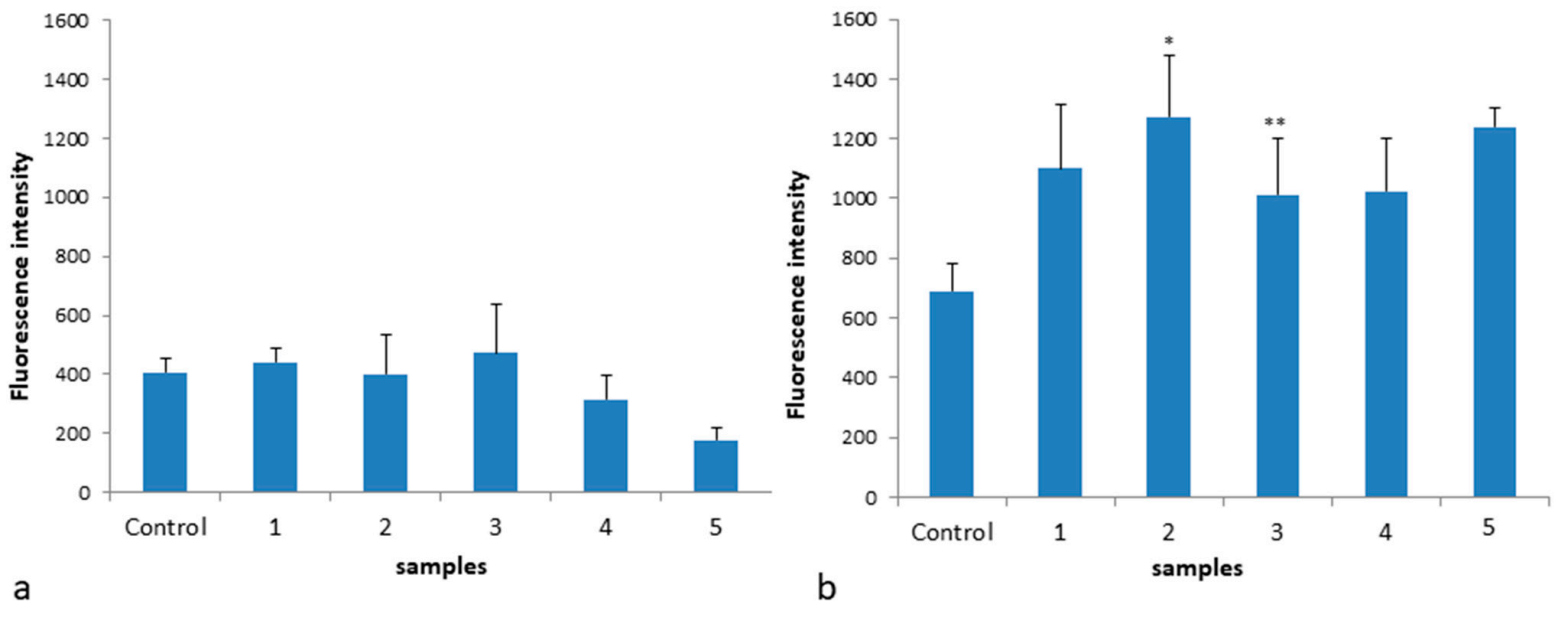
ROS production after treatment with (**1**) to (**5**) (**a**) at the 24th h and (**b**) at the 72nd h. Non-treated HepG2 cells were used as control. Bars are mean + SEM, n = 3. * *p* < 0.05; ** *p* < 0.01; asterisks indicate a significant difference between the sample and non-treated control.

It has been established that the cytotoxicity and genotoxicity of NPs are determined by their absorption and the generation of ROS [71]. To explore whether AgNPs induce oxidative stress, potentially enhancing their effectiveness as a vehicle for MBH and MA by increasing cell susceptibility to therapeutic effects, we measured reactive oxygen species (ROS) levels 24 and 72 h after treatment with all tested substances. As shown in Figure 8, AgNPs did not significantly increase ROS production in HepG2 cells at 24 h. However, at the 72 h time point, ROS levels were significantly elevated compared to untreated control cells. Among the treatments, substances (**2**) and (**5**) induced the highest ROS production, while substance (**3**) (AgNPs alone) resulted in the lowest ROS induction. This pattern, however, does not directly align with the cytotoxicity results, where AgNPs exhibited the highest cytotoxic effects. This discrepancy suggests that the cytotoxic effects of AgNPs are not solely dependent on ROS generation. Instead, the potential of AgNPs as a drug delivery vehicle for MBH and MA may rely on additional mechanisms, such as enhancing the bioavailability of the loaded compounds and promoting their sustained release, which can ultimately improve therapeutic efficacy without primarily relying on oxidative stress pathways.

### 2.6. Genotoxicity Testing of the Nanoformulations as Drug Delivery Vehicles for MBH and MA

**Figure 9:**
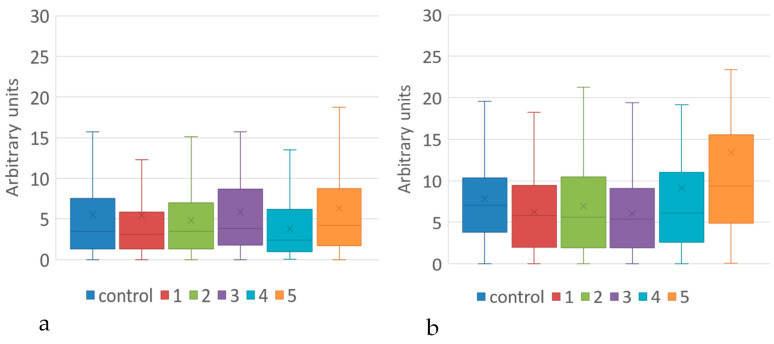
Genotoxic effects of the tested compounds **1** to **5** on HepG2 cells: DNA integrity across (**a**) 24th and (**b**) 72nd h. The genotoxic effects of compounds (**1**) to (**5**) were assessed using the neutral Comet Assay. HepG2 cells were cultured and treated at IC_50_ concentrations. DNA integrity was analyzed based on Olive Moment (OM) values, with data represented as (**a**) for 24 h treatments and (**b**) for 72 h treatments. Results are presented as box–whisker plots, reflecting three independent experiment repetitions.

The genotoxicity of compounds (**1**) to (**5**) was also evaluated. The results are presented in Figure 8 as box–whisker plots of the Olive Moment parameter. Following 24 h of treatment at IC_50_ concentrations (as detailed in Table 1), no genotoxic effects were detected in HepG2 cells (Figure 9a), indicating that DNA integrity remained intact. This suggests that neither the nanoparticles nor the tested compounds induced significant DNA damage during the initial exposure period.

In contrast, after the 72nd h of the treatment, a marked genotoxic effect was observed, particularly for compound (**5**) (Figure 9b). A slight increase in DNA strand breaks characterized this effect relative to the control group and other treatment conditions. The observed effect may stem from the synergistic interaction between AgNPs and the MA’s structural components, which could potentiate genotoxicity over time. These findings underscore the importance of considering the chemical composition of nanoparticle-based systems and the temporal dynamics of their interaction with biological systems. Prolonged exposure to such materials may compromise genomic stability, raising potential safety concerns for extended treatment duration applications.

This evaluation provides a basis for understanding the safety parameters of the tested compounds and transitions logically to the next chapter, where molecular docking studies are employed to explore the biological interactions of compounds (**1**) to (**5**).

### 2.7. Molecular Docking Simulations

The biological properties of (**1**) to (**5**) were further explored through molecular modeling. We limited the simulation of the nanoparticle structure to only a region consisting of two fructose molecules. Employing in silico docking simulations via AutoDock 4.2 and AutoDock Tools 1.5.6, the molecular interactions of these compounds with the VEGFR2 protein (PDB ID: 4AGD) were examined. The optimized molecular structures of the compounds were docked with VEGFR2 to evaluate their binding affinities, providing a comprehensive understanding of their potential biological effects. This computational approach was driven by the promising activity and safety potential of AgNPs demonstrated in experimental studies; by visualizing the key interaction patterns and identifying critical binding sites, the docking analysis aimed to validate and support the experimental findings. Such an integrative strategy offers more profound insights into the mechanism of action, enhancing the predictive accuracy of the therapeutic potential of these compounds.

**Figure 10:**
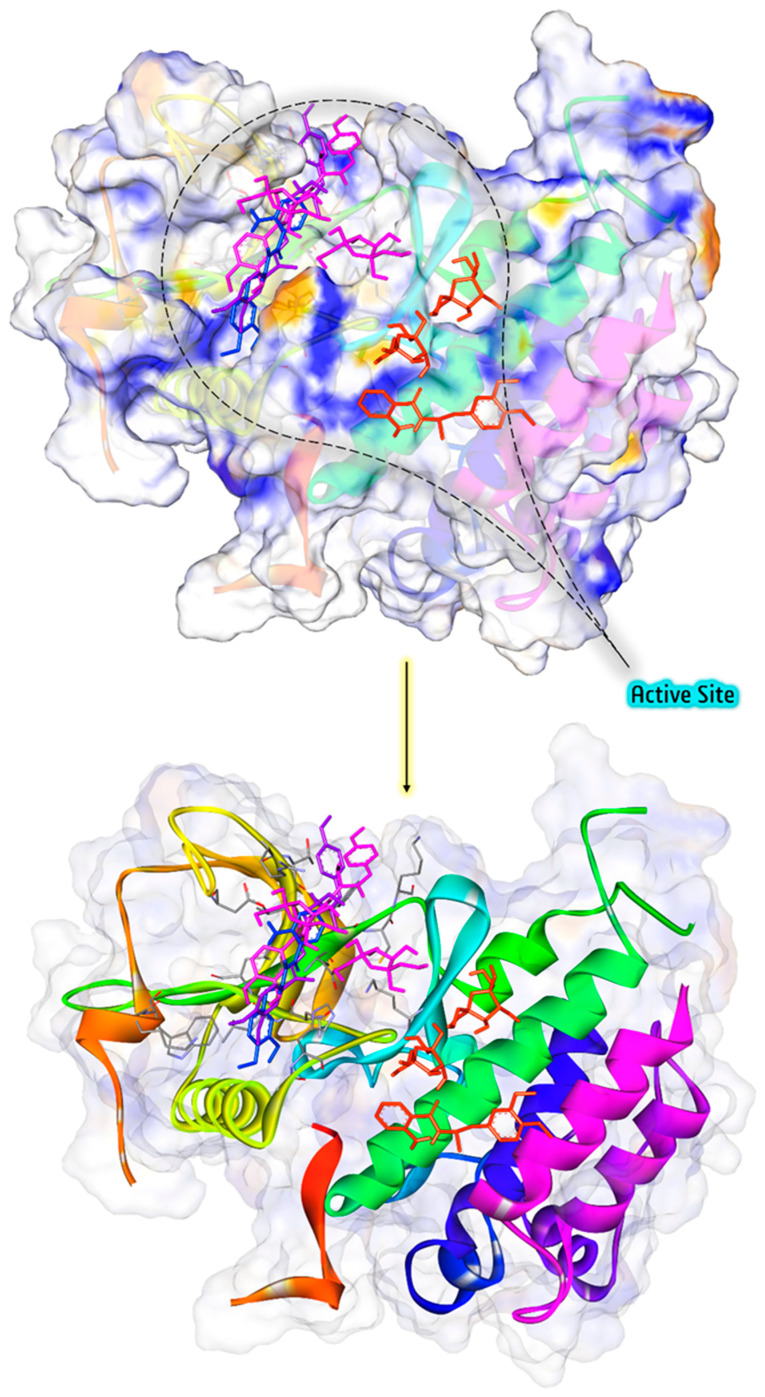
The docking perspective of (**1**) to (**5**) in the interaction with VEGFR2 protein.

The results of the docking analysis provide valuable insights into the binding interactions between the 4AGD protein and the tested compounds. These interactions’ calculated binding free energies were −6.80, −5.91, −6.83, −7.47, and −6.59 kcal/mol, respectively. These results suggest that the trend of the inhibitory activity of compounds against the target protein is as follows: (**5**) > (**3**) > (**2**) > (**4**) > (**1**). Notably, this observed pattern is consistent with the cytotoxicity data, further supporting the correlation with binding affinity. Moreover, the docking analysis underscores the superior efficacy of (**5**) in inhibiting cellular growth compared to the other tested agents. This enhanced potency identifies it as a promising candidate for further exploration in therapeutic strategies. The studied compounds demonstrate significant interactions within the target protein’s active site, as depicted in Figure 10.

**Figure 11:**
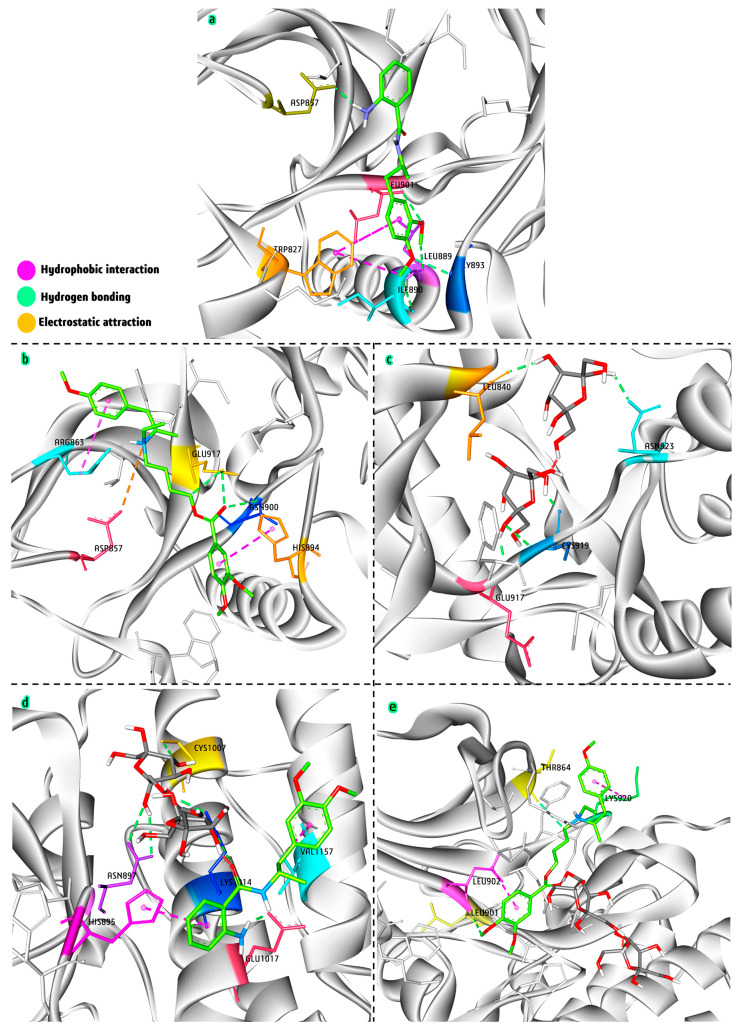
The 3D docking diagram of (**2**) (**a**), (**1**) (**b**), (**3**) (**c**), (**5**) (**d**), and (**4**) (**e**) in the interaction with VEGFR2 protein.

A comprehensive visualization of these interactions is provided in Figure 11, which showcases detailed 3D interaction diagrams of the compounds during their binding process with the 4AGD protein.

The findings reveal that AgNPs engage with the receptor primarily through hydrogen bonding, underscoring the critical role of these interactions in stabilizing the complex. In contrast, the binding behavior of (**1**) to (**5**) exhibits a more diverse interaction profile. These systems rely predominantly on hydrophobic interactions in addition to hydrogen bonding. This dual-mode interaction strategy highlights the complex nature of ligand–receptor binding, where both polar and non-polar forces contribute to the overall stability and specificity of the molecular complex.

## 3. Materials and Methods

All solvents and reagents and MBH hydrochloride were purchased from Merck (Merck KGaA, Darmstadt, Germany). MA (**2**) was characterized using IR, ^1^H-NMR, and ^13^C-NMR. The melting point was measured on a Kruss M5000 melting point meter (A.Krüss Optronic GmbH, Hamburg, Germany). IR spectra were determined on a VERTEX 70 FT-IR spectrometer (Bruker Optics, Ettlingen, Germany). ^1^H-NMR and ^13^C-NMR spectra were recorded on a Bruker Avance III HD 500 spectrometer (Bruker, Billerica, MA, USA) at 500 MHz (^1^H-NMR) and 125 MHz (^13^C-NMR), respectively. Chemical shifts are given in relative ppm and were referenced to tetramethylsilane (TMS) (δ = 0.00 ppm) as an internal standard; the coupling constants are indicated in Hz. The NMR spectra were recorded at room temperature (ac. 295 K). The purity was determined by TLC using several solvent systems of different polarities. TLC was carried out on precoated 0.2 mm Fluka silica gel 60 plates (Merck KGaA, Darmstadt, Germany), using chloroform/n-hexane/methanol/acetone = 4:4:1:1 as a chromatographic system.

### 3.1. Synthetic Protocol

#### 3.1.1. Synthesis of MA (**2**) (Scheme 1) [72]

A mixture of isatoic anhydride (**9**) (1.63 g, 10 mmol) and 1-(3,4-dimethoxyphenyl)propan-2-amine (**10**) (initially prepared from 1-(3,4-dimethoxyphenyl)propan-2-one) [73,74] (2.93 g, 15 mmol) dissolved in dichloromethane (30 mL) was stirred overnight at rt. The resulting solution was filtered on neutral Al_2_O_3_ and concentrated. Spectral data confirmed the structure of the obtained 2-amino-N-(1-(3,4-dimethoxyphenyl)propan-2-yl)benzamide (MA, **2**): m.p. 152.8 °C, ^1^H NMR (500 MHz, CDCl_3_) δ 1.14 (d, *J* = 6.7, 3H, CH_3_), 2.70–2.80 (m, 2H, CH_2_), 3.75 (s, 3H, OCH_3_) 3.78 (s, 3H, OCH_3_), 4.32 (dq, *J* = 13.4, 6.7 Hz, 1H, CH), 5.51 (broad s, 1H, NH_2_), 5.86 (d, *J* = 7.9 Hz, 1H, NH), 6.56–6.59 (m, 1H, Ar), 6.66–6.68 (m, 3H, Ar), 6.74 (d, *J* = 5, 1H, Ar), 7.11–7.15 (m, 2H, Ar); ^13^C NMR (126 MHz, CDCl_3_) δ 168.49, 148.86, 147.72, 147.46, 132.25, 130.37, 126.98, 121.59, 117.91, 117.43, 117.07, 112.62, 111.18, 55.91, 55.84, 46.16, 41.66, 19.99; FT-IR, cm^−1^: 3467 ν_as_ (-N-H, -NH_2_), 3371 ν_s_ (-N-H, -NH_2_), 3283 ν (-N-H, >NH-amide), 3067, 3055, 3033 ν (Csp^2^-H, -Ph), 2998 ν_as_ (Csp^3^-H, -OCH_3_), 2972 ν_as_ (Csp^3^-H, -CH_3_), 2935, 2914 ν_as_ (Csp^3^-H, -CH_2_-), 2874 ν_s_ (Csp^3^-H, -CH_3_), 2838 ν_s_ (Csp^3^-H, -OCH_3_), ν_s_ (Csp^3^-H, -CH_2_-), 1626 ν (>C=O), secondary amide I, 1584 ν (C-C=C, -Ph1,2,4), 1539, 1514 δ (N-H) and ν (C-N), trans-secondary amide II, 1463 ν (C-C=C, -Ph_ortho_), ν (C-C=C, -Ph1,2,4), δ_as_ (-CH_3_), δ_as_ (-OCH_3_), 1447 δ_s_ (-OCH_3_), 1416 ν (C-C=C, -Ph1,2,4), 1358 δ_s_ (-CH_3_), 1300 ν (C-C=C, -Ph_ortho_), ν (C-N), secondary amide III, 1256 δ (-Csp^2^-H, -Ph1,2,4), 1157 δ (-Csp^2^-H, -Ph_ortho_), ρ(-CH-)/ν (N-C) in –NH-CH-, 1139 δ (-Csp^2^-H, -Ph1,2,4), 1029 δ (-Csp^2^-H, -Ph_ortho_); HRMS electrospray ionization (ESI) *m*/*z* calcd. for [M+H]^+^ C_18_H_23_O_3_N_2_^+^ = 315.17032, found 315.16956 (mass error ∆m = −2.41 ppm).

**Scheme 1:**
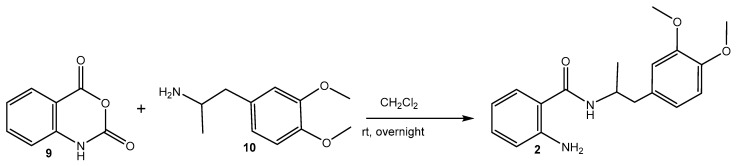
Reaction pathway for the synthesis of MA (**2**).

#### 3.1.2. Synthesis of Fructose-Assisted Ag NPs (**3**)

A total of 1.25 g (0.007 mol) fructose was dissolved in 25 mL water and refluxed for 5 min. Then, 0.63 mL of 0.01 M AgNO_3_ solution was added, and the mixture was kept at boiling. In about 5 min, the solution turned pale yellow, indicating the formation of **6**.

#### 3.1.3. Synthesis of MBH-Loaded AgNPs (**4**) and MA-Loaded Ag NPs (**5**)

A total of 1.25 g (0.007 mol) fructose was dissolved in 25 mL water and refluxed for 2 min; then, 0.63 mL of 0.01 M AgNO_3_ solution was added. Then, MBH (**1**) or MA (**2**) was added in a concentration of 1 mg/mL. The solution’s color changed to a light yellow after roughly 5 min of reflux, signifying the formation of (**4**) or (**5**).

### 3.2. Characterization of the Ag NPs: Analytical Techniques

After NP preparation, the solution was used for UV-Vis, transmission electron microscopy (TEM), dynamic light scattering (DLS), and zeta potential measurements.

#### 3.2.1. TEM

The morphology, shape, and size of the nanoparticles were investigated by TEM (Talos F200C G2, Thermo Scientific, Landsmeer, The Netherlands). The nanoparticle suspension was added dropwise onto a formvar/carbon-coated copper grid, and then the TEM observation of the samples was performed at an operating voltage of 200 kV.

#### 3.2.2. DLS and Zeta Potential

DLS measurements were performed on a Brookhaven BI-200 goniometer (Brookhaven Instrumetns Corporation, Holtsville, NY, USA) with vertically polarized incident light at a wavelength l = 632.8 nm supplied by a He–Ne laser operating at 35 mW and equipped with a Brookhaven BI-9000 AT digital autocorrelator (Brookhaven Instrumetns Corporation, Holtsville, NY, USA). The scattered light was measured for dilute aqueous dispersions in the concentration range 0.056–0.963 mg mL^−1^ at 25, 37, and 65 °C. Measurements were made at angles y in the range of 50–130°. The system allows measurements of ζ-potential in the range from −200 mV to +200 mV. All analyses were performed in triplicate at 25 °C.

#### 3.2.3. UV-Vis Spectra

UV-Vis spectra were recorded between 320 and 800 nm using a Cary-60 UV-Vis spectrophotometer (Agilent Technologies, Santa Clara, CA, USA). Solution spectra were obtained by measuring the absorption of the prepared nanoparticle dispersions in a quartz cuvette with a 1 cm optical path.

#### 3.2.4. FTIR Spectra

IR spectra were determined on a VERTEX 70 FT-IR spectrometer (Bruker Optics, Ettlingen, Germany). The spectra were collected from 600 cm^−1^ to 4000 cm^−1^ with a resolution of 4 nm and 32 scans. The instrument was equipped with a diamond attenuated total reflection (ATR) accessory. The IR spectra were analyzed with the OPUS-Spectroscopy Software, Bruker (Version 7.0, Bruker, Ettlingen, Germany).

### 3.3. Molecular Docking

An in silico docking analysis was carried out to explore the cytotoxic potential of the compounds further, complementing the results of in vitro experiments. The computational study utilized AutoDock 4.2 [75] and AutoDock Tools 1.5.6 [76], renowned molecular docking and binding analysis tools. The high-resolution crystallographic structure of the Vascular Endothelial Growth Factor Receptor 2 (VEGFR2) protein (PDB ID: 4AGD) was obtained from the RCSB Protein Data Bank (www.rcsb.org), accessed on 19 December 2024. Optimization procedures were undertaken to prepare the 4AGD structure for docking simulations. This process involved the removal of non-essential atoms, ligands, and water molecules that could interfere with the accuracy of the docking process. To further enhance the precision of the simulation, polar hydrogens were added, and Kollman charges were assigned to the structure. These modifications ensured a more accurate representation of the protein’s binding environment, enabling a more reliable prediction of ligand–protein interactions. The structure of the compounds was obtained from the theoretical approach. This study employed computational techniques to construct and optimize the 3D structures of (**1**) and (**2**) and AgNPs functionalized with two fructose molecules and drugs. These molecular models were drawn using Gauss View 6.0 and optimized through the Gaussian 09W (Revision D.01, Gaussian, Inc.) software suite to facilitate docking simulations. The density functional theory (DFT) approach was utilized for geometry optimizations, explicitly employing the B3LYP method with the 6-311G(d,p) basis set for all atoms involved. To accurately identify the docking site, a grid box was established with a finely tuned spacing of 0.375 Å. The center of this grid was aligned with the active site at the coordinates corresponding to 4AGD, encompassing dimensions of 90 × 90 × 80. Docking simulations were conducted utilizing the Lamarckian Genetic Algorithm (LGA). Following the completion of the simulations, the resulting binding conformations were thoroughly examined using the Discovery Studio Visualizer 4.1 software to facilitate an in-depth analysis of the interactions. The docking procedure was meticulously carried out in alignment with the established protocols outlined in the published literature [77,78,79].

### 3.4. Cell Cultures

HepG2 cells, derived from a human liver carcinoma, were cultured in Dulbecco’s modified Eagle’s medium (DMEM) culture with 10% fetal bovine serum (FBS; Merck KGaA, Darmstadt, Germany). Cells grew in a humidified environment with 5% CO_2_ and 95% humidity at 37 °C; 0.05% trypsin and 0.02% EDTA solution (Merck KGaA, Darmstadt, Germany) were used to detach cells for in vitro experiments. For cytotoxicity experiments (WST-1 assay, ROS, phase-contrast morphology), the cells were seeded in 24-well plates with a concentration of 2.5 × 10^4^ cells/well. The cells were cultivated for 24 h and then treated with NPs.

### 3.5. Phase-Contrast Light Microscopy

Following a 24 and 72 h exposure to the studied NPs, phase-contrast observations were made to assess changes in cell morphology. Micrographs were taken at magnifications of 20× with a Zeiss microscope (Axiovert 25, Carl Zeiss, Jena, Germany) fitted with a digital camera.

### 3.6. WST-1 Assay

WST-1 was performed to evaluate cell viability and metabolic activity. At the end of incubation, the medium with NPs was removed, and the cells were washed with PBS. Further, a 300 μL medium with 30 μL WST-1 (tetrazolium salt 4-(3-(4-iodophenyl)-2-(4-nitrophenyl)-2 h-5-tetrazolium)-1,3-benzene disulfonate) was added to all wells. Following a 1 h incubation period in the dark, an ELISA reader Thermo Scientific Multiskan Spectrum (Thermo Scientific, Tokyo, Japan) was employed to estimate the optical density of the samples at 450 nm.

### 3.7. DCFA-DA Analysis

As described before, intracellular reactive oxygen species (ROS) production was measured using 2,7-dichlorofluorescin diacetate (DCFH-DA, Sigma-Aldrich, St. Louis, MO, USA). DCFH-DA is a non-fluorescent compound that passively enters the cell, reacts with ROS, and forms the highly fluorescent compound dichlorofluorescin (DCF). In brief, HepG2 cells (2.5 × 10^4^) were seeded in 24-well plates, allowing adherence. The next day, NPs with different concentrations were added to the cells and incubated for 24 h. Following respective exposure, cells were washed twice with PBS and incubated for 30 min in the dark in an FBS-free culture medium containing DCFH-DA (20 μM). Then, the DCFH-DA-containing medium was removed, the control (untreated) and treated cells were rinsed twice with PBS, and the fluorescence intensity of DCF was detected on a spectrofluorometer upon excitation at 485 nm and emission at 520 nm.

### 3.8. Genotoxicity Testing

The neutral Comet Assay evaluated the genotoxicity of (**1**) to (**5**). HepG2 cells were cultured in Dulbecco’s modified Eagle’s medium (DMEM) supplemented with 10% fetal bovine serum (FBS), 100 U/mL penicillin, and 10 µg/mL streptomycin, and maintained at 37 °C in a humidified atmosphere with 5% CO_2_. Following treatment with the compounds for 24 and 72 h, the cells were collected by centrifugation at 2800× *g* for 5 min and washed with phosphate-buffered saline (PBS) to remove unbound substances. The cells were then resuspended in PBS and mixed with low-gelling agarose at a final concentration of 0.7% (*w*/*v*). This agarose–cell mixture was spread onto precoated microscopic slides and covered with a coverslip; after solidification at 4 °C for 10 min, the coverslip was removed. The slides containing the agarose-embedded cells were incubated in lysis solution (1 M NaCl; 50 mM EDTA, pH 8.0; 30 mM NaOH; 0.1% N-laurylsarcosine) for 60 min to disrupt cell membranes and remove proteins. Following lysis, the slides were submerged in alkaline electrophoresis buffer (10 mM EDTA, pH 8.0; 30 mM NaOH) for 30 min to unwind double-stranded DNA. Electrophoresis was conducted at a voltage of 0.45 V/cm for 20 min to allow DNA fragments to migrate towards the anode. After electrophoresis, the slides were rinsed with distilled water to neutralize the alkali in the gel and dehydrated by incubating in 75% and then 95% ethanol for 5 min each before air-drying. The DNA was stained using SYBR Green I (Molecular Probes) before observation under an epi-fluorescent microscope equipped with a 450–490 nm bandpass filter at a magnification of 250×. Comet Assay data quantitation was performed using CometScore software 2.0 (TriTek Corporation, Sumerduck, Virginia, USA), focusing on parameters such as Olive Moment (OM), which integrates both tail length and intensity to assess genotoxicity comprehensively.

### 3.9. Statistical Analysis

The obtained data were analyzed using Microsoft Excel software version 10 (Microsoft, Redmond, WA, USA). The presented bars illustrated mean values of the MDCK and HepG2 cell viability/metabolic activity ± STDV. Probability levels of * *p* < 0.05, ** *p* < 0.01, and *** *p* < 0.005 were considered statistically significant after the performance of Student’s *t*-test.

## 4. Conclusions

MBH-loaded AgNPs and MA-loaded AgNPs were synthesized for the first time. The results of this study demonstrate the successful synthesis of AgNPs using fructose as a reducing and capping agent, leading to the formation of nanoparticles with controllable size and promising properties. The drug-loaded Ag NPs were characterized in vitro for their cytotoxic and genotoxic effect, compared to MBH, resp. MA, and plain AgNPs. Drug-loaded Ag NPs, on the other hand, showed better characteristics than MBH, or MA, itself.

Cytotoxicity testing revealed that AgNPs, particularly when combined with MA, exhibited concentration-dependent cytotoxic effects in HepG2 cells, with AgNPs alone showing the highest toxicity. Additionally, genotoxicity tests indicated no significant DNA damage at 24 h. Still, prolonged exposure to AgNPs in combination with MA resulted in slightly increased DNA strand breaks, highlighting the impact of treatment duration on genotoxicity.

Molecular docking studies provided further insights into the binding interactions between the compounds and the VEGFR2 protein, supporting the cytotoxicity data. The binding affinities suggested that the AgNPs loaded with MA (**5**) had more substantial interactions with the target protein, highlighting their potential as nanocarriers for mebeverine delivery. This computational analysis aligns with the experimental results and underscores the promising application of these formulations in enhancing drug delivery systems for IBS management.

Overall, the synthesized AgNPs show significant promise as drug delivery vehicles, with their combination with MBH and MA enhancing both bioavailability and efficacy. Their ability for targeted administration and low genotoxicity indicate that they may be developed into safe and effective medicines, warranting further investigation in vivo.

## Figures and Tables

**Table 1:** IC_50_ values of the tested compounds **1**–**5** against HepG2 cells at the 24th h and 72nd h.

Samples	IC_50_ at 24th h [µg/mL]	IC_50_ at 72nd h [µg/mL]
**1**	17.16	27.50
**2**	24.69	29.39
**3**	6.35	17.79
**4**	14.16	17.94
**5**	8.88	13.93

## Data Availability

The original contributions presented in the study are included in the article. Further inquiries can be directed to the corresponding authors.
